# Surface-mutagenesis strategies to enable structural biology crystallization platforms

**DOI:** 10.1107/S2059798324007939

**Published:** 2024-08-29

**Authors:** Martina Schaefer, Vera Pütter, André Hilpmann, Ursula Egner, Simon James Holton, Roman Christian Hillig

**Affiliations:** a Structural Biology, Nuvisan ICB GmbH, Muellerstrasse 178, 13353Berlin, Germany; b Protein Technologies, Nuvisan ICB GmbH, Muellerstrasse 178, 13353Berlin, Germany; UNSW Sydney, Australia

**Keywords:** surface modification, surface-entropy reduction, crystal packing, drug discovery, Aurora-C, BUB1, IRAK4, KRAS–SOS1, structure-based drug discovery, crystallization platforms

## Abstract

A key prerequisite for the successful application of protein crystallography in drug discovery is to establish a robust crystallization system for a new drug-target protein fast enough to deliver crystal structures when the first inhibitors have been identified in the hit-finding campaign or, at the latest, in the subsequent hit-to-lead process. The first crucial step towards generating well folded proteins with a high likelihood of crystallizing is the identification of suitable truncation variants of the target protein. In some cases an optimal length variant alone is not sufficient to support crystallization and additional surface mutations need to be introduced to obtain suitable crystals. In this contribution, four case studies are presented in which rationally designed surface modifications were key to establishing crystallization conditions for the target proteins (the protein kinases Aurora-C, IRAK4 and BUB1, and the KRAS–SOS1 complex). The design process which led to well diffracting crystals is described and the crystal packing is analysed to understand retrospectively how the specific surface mutations promoted successful crystallization. The presented design approaches are routinely used in our team to support the establishment of robust crystallization systems which enable structure-guided inhibitor optimization for hit-to-lead and lead-optimization projects in pharmaceutical research.

## Abstract 2

Successful crystallographic structure-based drug-discovery (SBDD) support of early-stage pharmaceutical research programs requires the timely establishment of a robust crystallization system for the target protein. This enables structure determination of early high-throughput screening or fragment-screening hits, in turn maximizing the impact of SBDD on hit optimization. Here, a collection of complementary case studies are presented that demonstrate typical strategies to achieve this goal.

## 1. Introduction

The key task of structural biology in pharmaceutical research is to deliver crystal structures of the target protein in complex with early hits, for example from high-throughput screening (HTS) or fragment-screening activities. The availability of binding-mode data is especially valuable at an early stage in the optimization of compounds, providing guidance for the computational and medicinal chemistry design teams and triggering new design ideas for subsequent optimization cycles. Historically, identifying and optimizing crystallization conditions was very time-consuming and protein crystal structures often came too late to contribute towards compound optimization. Over the last 25 years, crystallization success has increased massively, driven by a combination of factors including progress on the side of the crystallization experiment, such as the development and commercialization of sparse-matrix screens (see, for example, Jancarik & Kim, 1991 ▸; Page & Stevens, 2004 ▸) and the development of nanodrop pipetting robots and imaging robots (see, for example, Newman *et al.*, 2008 ▸). Additionally, an improved understanding of construct design for structural biology has been instrumental in improving crystallization success rates. Drug-target proteins often contain multiple domains, of which only one is the actual target of a drug-discovery project. Whilst the generation of protein constructs containing only the domain(s) of interest can significantly increase the technical chances of success, the selection of the correct N- and C-termini is crucial. Termini that are too long and flexible may prevent crystal lattice formation, while termini that are too short may prevent the domain from folding correctly (see, for example, Malawski *et al.*, 2006 ▸). The design of suitable truncation-length variants is typically supported by a spectrum of data including scientific experience/know-how, limited proteolytic digest experiments of the full-length protein and *in silico* modelling of the domain structures of the target protein. Typically, soluble expression levels and subsequent thermal stability measurements of the different truncation-length variants are used to guide the selection of a subset of constructs for further characterization (see, for example, Bandeiras *et al.*, 2008 ▸).

Whilst the generation of truncated proteins with suitable construct boundaries is often a very powerful strategy to support protein expression and purification, some challenging targets remain resistant to crystallization efforts despite the availability of protein of high quality and high purity. For these targets, additional modification of the protein may further encourage crystallization. Several strategies have been described whereby specific surface residues are modified to further increase the likelihood of successful crystallization. One approach is to stabilize intrinsically flexible parts of a protein by targeting post-translational modification sites such as the phosphorylation sites in the activation segment of protein kinases [reviewed in Müller (2017 ▸); see also Bandeiras *et al.* (2008) as an example]. Another is to mutate clusters of surface residues with inherent high entropy [the surface-entropy reduction (SER) approach; Derewenda (2004 ▸)]. In the SER approach, surface clusters of lysine, arginine, glutamate and glutamine residues with many rotational bonds with inherent high surface entropy are targeted (Derewenda, 2004 ▸; Derewenda & Vekilov, 2006 ▸). Such residues may prevent crystal packing as they would lose too much entropy when forced to adopt only a single rotamer conformation when engaging in a crystal contact. The approach consists of manually inspecting the primary sequence or a homology model of the target protein for surface patches of adjacent lysine, arginine, glutamate and glutamine residues, and mutating some of these residues to shorter, less entropically active residues such as alanine. Subsequently, it has been reported that mutating high-entropy surface residues to tyrosine or threonine residues can also be very beneficial (Cooper *et al.*, 2007 ▸). Tyrosine and threonine residues are also typically rigid, with a small number of preferred rotamers. However, in contrast to alanine residues, they can contribute both a hydrophobic interaction surface as well as a hydrogen-bond donor and acceptor that may be beneficial for forming new crystal contacts.

Finally, another alternative, and complementary, surface-modification approach to promote crystallization can be applied in cases where the target protein cannot be crystallized but crystal structures have been reported of a closely related isoform. Surface residues contributing to key crystal lattice contacts in the crystallized isoform are identified by manual inspection and their sequence conservation in the noncrystallizing isoform is analysed. Nonconserved residues in the noncrystallizing isoform can then be mutated to those from the crystallized isoform to promote such contacts and hence crystallization of the noncrystallizing isoform. Literature examples of this approach are often not clearly assigned as such, but the structure determination of the kinase domain of HER2 upon the introduction of a triple surface mutation (to the corresponding residues found in the closely related and well crystallizing protein kinase EGFR; Aertgeerts *et al.*, 2011 ▸) and the structure determination of the difficult-to-crystallize kinase CSNK1α upon the mutation of three surface residues (to those found in the easily crystallizing isoform CSNK1δ; Minzel *et al.*, 2018 ▸) are probably cases in point.

Typically, we design and test crystallization constructs in two waves. Initial ‘first-generation’ constructs aim to scout and identify construct boundaries suitable for the recombinant production of protein for crystallization. Crystallization experiments with these constructs offer the first opportunity to evaluate their suitability for structural biology experiments. For those targets that resist crystallization, either with no suitable crystals or with only poorly diffracting crystals, a further set of constructs are designed and tested. These ‘second-generation’ constructs typically focus on the introduction of surface mutants designed to further increase the likelihood of successful crystallization. These mutations are typically introduced into the most promising first-generation truncation constructs. The utmost care must be taken during the design of these mutations to ensure that residues close to the site of interest remain undisturbed.

In this contribution, we present and discuss a collection of case studies for which the combination of both domain truncations and additional surface mutagenesis were required to successfully establish the crystallization of challenging protein targets.

## 2. Materials and methods

### 2.1. Crystallization, data collection and structure determination of the Aurora-C–INCENP complex via the SER approach

The set of eight expression constructs for human Aurora kinase C (Aurora-C; UniProt ID Q9UQB9) used in this study are shown in Table 1 ▸. Of these, AurC_8 ultimately enabled crystallization and structure determination. Sf9 insect cells were co-infected with construct AurC_8 (with the triple mutation R195A, R196A, K197A and an N-terminal His tag) and a construct of untagged human inner centromere protein (INCENP; UniProt ID Q9NQS7, residues 834–891). The cells were lysed, supplemented with the high-affinity inhibitor staurosporine and purified via Ni^2+^–IMAC. The combined fractions of Aurora-C and co-purified INCENP peptide were re-supplemented with inhibitor, concentrated and further purified by gel-filtration chromatography and a final cation-chromatography step (Mono S; start buffer 20 m*M* MES pH 6.5, 1 m*M* DTT; elution buffer 20 m*M* MES pH 6.5, 1 *M* NaCl, 1 m*M* DTT; 0–50% gradient in 20 column volumes). The yield was 0.5–1.0 mg per litre of insect culture. The final sample was concentrated to 10 mg ml^−1^ using an Amicon Ultra-4 centrifugal filter device.

An initial crystallization condition (reservoir condition 0.1 *M* bis-Tris pH 5.5, 0.2 *M* ammonium sulfate, 25% PEG 3350) was identified in a sparse-matrix screen. Optimization of this condition to a final reservoir composition of 0.1 *M* bis-Tris pH 5.5, 0.025–0.050 *M* ammonium sulfate, 9–12% PEG 3350 resulted in crystals that belonged to space group *C*222_1_ with two Aurora-C–INCENP complexes in the asymmetric unit. A crystal was cryoprotected by transfer into mother liquor supplemented with 15% glycerol. A data set to 2.8 Å resolution (Table 2 ▸) was processed using *CrystalClear* (Rigaku Corporation). The structure was solved by molecular replacement using *Phaser* (McCoy *et al.*, 2007 ▸) with Aurora-B–INCENP as the search model (one monomer of PDB entry 2bfy) and refined using *REFMAC*5 (Murshudov *et al.*, 2011 ▸) from the *CCP*4 suite (Agirre *et al.*, 2023 ▸). There was only a small amount of positive difference density, which was not sufficient to place the ligand in the ATP-binding site. This observation is consistent with the absence of inhibitor in the final chromatography step and the crystallization experiments. The data-collection and refinement statistics are shown in Tables 2 ▸ and 3 ▸, respectively. The PDB accession code is 9esa.

### 2.2. Crystallization, data collection and structure determination of protein kinase IRAK4 via the SER approach

A set of 19 length and mutation variants of the kinase domain of IRAK4 (IRAK4; UniProt ID Q9NWZ3) depicted in Table 4 ▸ were expressed as GST-tagged proteins in SF9 insect cells following the cloning, expression and purification protocols described previously for IRAK4_6 (Bothe *et al.*, 2024 ▸). In brief, a total of six of the 19 different IRAK4 expression constructs designed in the course of this study (Table 4 ▸, length variants and mutated versions) were purified via affinity chromatography, tag cleavage using thrombin, ion-exchange chromatography and final size-exclusion chromatography. The constructs were concentrated to 10–15 mg ml^−1^ using Amicon Ultra-4 centrifugal filter devices. Crystallization screening comprised a set of ten commercially available screens and one fine screen around the known conditions for a related kinase. In addition, the protein was also incubated with either staurosporine (a known pan-kinase inhibitor) or IRAK4 inhibitors identified by HTS (Bothe *et al.*, 2024 ▸). Crystals were obtained for all purified proteins except for construct IRAK4_5.

Final crystals of the SER variant IRAK4_6 were grown using the vapour-diffusion method with drops consisting of equal volumes of IRAK4_6 (∼10 mg ml^−1^ in 50 m*M* HEPES pH 7.6, 250 m*M* NaCl, 10% glycerol, 2 m*M* DTT) and reservoir solution (see below). Both co-crystallization and back-soaking methods were established to generate co-complex structures. In the co-crystallization experiments, inhibitors (100 m*M* stock solution in DMSO) were added to the protein to a final concentration of 2 m*M*. The complexes were incubated for 2 h on ice and crystallization was performed at 4°C using the vapour-diffusion method in hanging drops. Crystallization drops were set up using equal volumes of protein solution and reservoir solution [0.1 *M* sodium acetate buffer pH 4.9, 1.5–1.7 *M* ammonium citrate, 0.02 *M* hexammine cobalt(III) chloride]. Crystals with dimensions of 0.1–0.2 mm appeared within 1–3 days at 20°C. In a back-soaking experiment, crystals of a target protein are first grown in the presence of a tool inhibitor. These crystals are then used to soak out the tool compound and soak in the inhibitor of interest. For IRAK4, the tool compound (a 100 m*M* stock solution in DMSO) was added to the protein to a final concentration of 5 m*M* and the complex was incubated for 2 h on ice. Crystallization was performed by vapour diffusion in hanging drops using equal volumes of protein solution and reservoir solution (0.1 *M* sodium acetate buffer pH 4.9, 2.130–2.145 *M* sodium malonate) and the subsequent addition of IRAK4 seed crystals (previously obtained with the same tool compound). Crystals of IRAK4 with the tool compound grew after 1–3 days at 20°C to a final size of ∼0.1–0.3 mm. These crystals were then washed three times in reservoir solution overnight to wash out the tool compound. The inhibitors of interest (100 m*M* stock solutions in DMSO) were diluted with reservoir solution to a final concentration of 5 m*M* and the washed crystals of IRAK4 were soaked in this solution for 3–4 days at 20°C.

Data collection, structure determination and refinement has been described previously (Bothe *et al.*, 2024 ▸) and the structures have been deposited with the associated PDB codes 8atb, 8atl, 8atn, 8br6 and 8br7.

### 2.3. Crystallization, data collection and structure determination of a SER variant of the protein kinase BUB1

The mutated BUB1 kinase domain [construct BUB1_6 containing residues 726–1085 in which the seven C-terminal residues were mutated from ^1079^ECKRSRK^1085^ (wild-type BUB1) to ^1079^DYAPSYA^1085^; Table 5 ▸] was expressed and purified as described previously (Siemeister *et al.*, 2019 ▸). Crystals of this protein were grown at 4°C using the sitting-drop method by mixing 1 µl protein solution (concentrated to 14.7 mg ml^−1^ using an Amicon Ultra 15 centrifugal filter device) with 1 µl well solution (100 m*M* Tris–HCl pH 7.26, 200 m*M* MgCl_2_, 20% PEG 3350, 5% glycerol). A single crystal was briefly immersed in cryoprotection solution consisting of mother liquor supplemented with 20% glycerol and then flash-cooled in liquid nitrogen. X-ray data were collected on the Helmholtz-Zentrum Berlin beamline 14.1 at a wavelength of 0.91814 Å using a PILATUS detector. Data were integrated, scaled and merged using *XDS* (Kabsch, 2010 ▸) and *AIMLESS* (Evans & Murshudov, 2013 ▸). The structure was solved by molecular replacement using *Phaser* (McCoy *et al.*, 2007 ▸). The model was refined by iterative manual and maximum-likelihood refinement using *Coot* (Emsley *et al.*, 2010 ▸) and *REFMAC*5 (Murshudov *et al.*, 2011 ▸).

### 2.4. Crystallization of the KRAS^G12C^–SOS1 complex via surface modifications of KRAS

A set of eight KRAS^G12C^ variants with seven different surface mutations (Table 6 ▸) and the catalytic domain of human SOS1 were expressed and purified as described previously for the KRAS construct KRAS^G12C^_5 (Hillig *et al.*, 2019 ▸), which contains the triple surface mutation D126E, T127S, K128R. To arrive at this successful construct, the surface-mutant variants of human KRAS were introduced into two different C-terminal truncation variants of KRAS (UniProt ID P01116-2; amino acids 1–169 and 1–166, respectively), expressed on a small scale with an N-terminal His_10_ tag, purified via Ni–NTA chromatography followed by tag cleavage and gel filtration (in buffer consisting of 20 m*M* HEPES pH 8.0, 150 m*M* NaCl, 1 m*M* DTT), and concentrated to 40 mg ml^−1^ (this and all further concentration steps were performed using Amicon Ultra-15 centrifugal filter devices). The catalytic domain of SOS1 (UniProt ID Q07889, amino acids 564–1049, with an N-terminal His_10_ tag and a TEV cleavage site) was purified via Ni–NTA chromatography followed by tag cleavage and gel filtration (25 m*M* Tris–HCl pH 7.5, 100 m*M* NaCl, 1 m*M* DTT) and concentrated to 44 mg ml^−1^. Three mutants, KRAS^G12C^_1, KRAS^G12C^_5 and KRAS^G12C^_8, were purified on a large scale and the respective complexes with SOS1 were formed by the release and removal of GDP from KRAS in the presence of a threefold molar excess of SOS1, followed by gel filtration and concentration of the KRAS–SOS1 complex to 15 mg ml^−1^, as described for KRAS_5 in Hillig *et al.* (2019 ▸). The crystals obtained for KRAS^G12C^_5 were optimized and enabled the structure determination of five KRAS^G12C^–SOS1 inhibitor co-complexes, as reported by Hillig *et al.* (2019 ▸) (PDB entries 6epl, 6epm, 6epn, 6epo and 6epp).

## 3. Results and discussion

### 3.1. Structure of the Aurora-C–INCENP complex

After initial attempts to express different truncation variants of the kinase domain of Aurora-C in *Escherichia coli* did not produce soluble protein, a further six truncation variants (combinations of three different N-termini starting at either residues 13, 28 or 35 and two different C-termini ending at residues 301 or 309; Table 1 ▸) were designed based on the expression constructs used to crystallize the closely related kinase Aurora-A and careful analysis of which terminal residues were ordered in the respective Aurora-A structures (Nowakowski *et al.*, 2002 ▸; Cheetham *et al.*, 2002 ▸). The six constructs were expressed using baculovirus in insect cells, all in parallel, with hexahistidine tags at either the N-terminus or the C-terminus. The best expression levels were observed for the constructs 13–301 and 13–309. However, low yields after gel filtration, coupled with protein precipitation during concentration, prevented extensive crystallization experiments. We therefore switched to co-expressing N-terminally histidine-tagged Aurora-C (residues 13–309) with a peptide comprising of residues 835–892 of the IN-box segment of the inner centromere protein (INCENP) activator, which had previously been co-crystallized with Aurora-B (Sessa *et al.*, 2005 ▸) and which had also been reported to bind to Aurora-C (Li *et al.*, 2004 ▸). The resulting Aurora-C–INCENP complex indeed eluted as a complex from the IMAC column, but again the sample precipitated during subsequent concentration. This was overcome by the addition of a high-potency inhibitor (either staurosporine or an in-house Aurora-C inhibitor, data not shown) both before and after the IMAC purification step. The majority of the Aurora-C protein now eluted from the gel-filtration column with the expected molecular weight. The protein yield was significantly reduced in the absence of INCENP, indicating that both the INCENP peptide and a high-potency inhibitor were required to maintain protein solubility. Whilst the Aurora-C–INCENP–inhibitor complex could now be concentrated to 10 mg ml^−1^, extensive crystallization screening did not identify any hits.

We noted that in a published Aurora-A crystal structure (PDB entry 1mq4), a phosphate ion from the crystallization buffer mimicked a phosphorylated threonine in the activation segment and may have facilitated crystallization by stabilizing this conformationally flexible loop. We therefore designed a triple-aspartate mutation (S193D, T198D, T202D) in which all three Aurora-C activation-segment phosphorylation sites were replaced with negatively charged residues, thus mimicking the phosphorylated and fully activated form of Aurora-C. This new construct resulted in an Aurora-C–INCENP complex which expressed and purified with higher yield than the wild-type protein, but again did not crystallize.

We therefore stopped work with this triple-aspartate mutation and instead introduced the surface-entropy reduction (SER) triple mutation (R195A, R196A, K197A) into the activation segment of Aurora-C. While we usually design, clone and express five to ten SER mutants in parallel to increase the likelihood of success (see the case studies reported below), we selected only this one SER triple mutant here because it represents the only cluster of Arg, Lys, Gln or Glu residues in the activation loop of Aurora-C. The Aurora-C–INCENP complex now eluted in two adjacent peaks in the final Mono S chromatography step, and fractions from both peaks yielded crystals in several conditions of an initial screen. Optimization of an initial hit condition yielded crystals which diffracted to 2.8 Å resolution. The structure could be solved by molecular replacement using an Aurora-B–INCENP structure (PDB entry 2bfx) as a search model (Tables 2 ▸ and 3 ▸).

**Figure 1:**
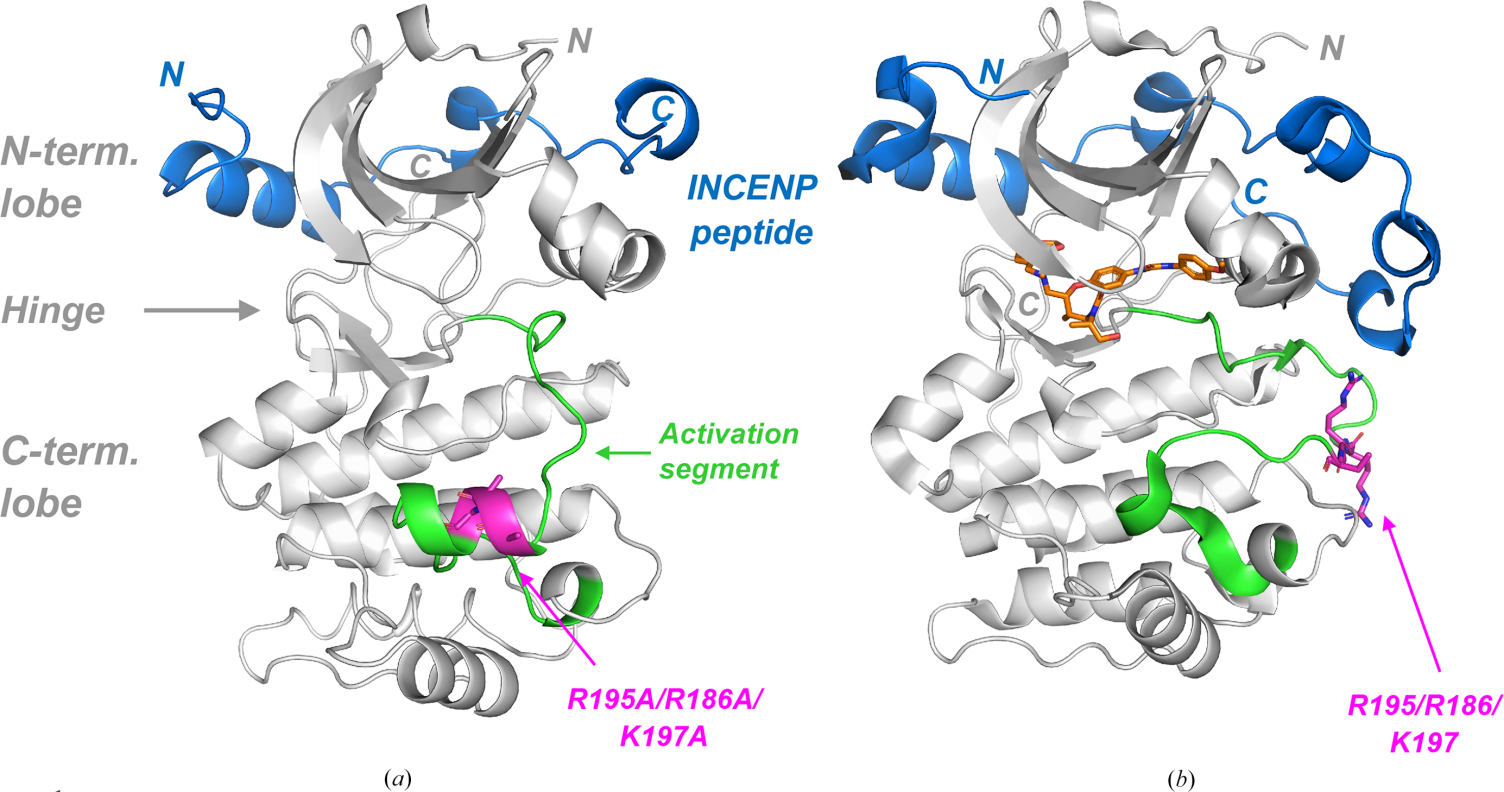
Overall fold of Aurora-C and location of the triple SER mutation. (*a*) Overall complex (chain *A*) with the INCENP peptide (residues 835–892) in blue and the activation segment (^184^DFG…PPE^209^) in green. The three SER mutations, R195A, R196A and K197A, are shown as stick models with the C atoms in magenta. For comparison, (*b*) shows a structure of Aurora-C (PDB entry 6gr8) with an inhibitor (orange), a longer INCENP peptide (834–903) and without the three SER mutations (residues Arg195, Arg196 and Lys197 shown with C atoms in magenta).

The final structure contains two Aurora-C–INCENP complexes in the asymmetric unit. Both Aurora-C chains feature the same overall conformation. Aurora-C adopts the typical protein kinase fold, with an N-terminal and a C-terminal lobe connected by the hinge region (Fig. 1 ▸*a*). Based on the absence of the salt bridge between Lys72 in the ATP site and Glu91 in helix C (8.9 Å distance), Aurora-C crystallized in an inactive conformation. The INCENP peptide wraps around the N-terminal lobe, forming extensive interactions with the kinase domain. Similar interactions between an INCENP peptide and a kinase have also been described for Aurora-B (Sessa *et al.*, 2005 ▸), as well as in two Aurora-C structures (PDB entries 6gr8 and 6gr9; Abdul Azeez *et al.*, 2019 ▸) which were published after this work had been completed. A comparison of one of these Aurora-C–INCENP structures (PDB entry 6gr8; Fig. 1 ▸*b*) with our Aurora-C–INCENP complex (Fig. 1 ▸*a*) reveals that the overall fold and the binding mode of the INCENP peptide around the N-terminal lobe are conserved. However, the two structures feature different conformations of the activation segment. In particular, the section with the SER triple mutant (magenta in Fig. 1 ▸*a*) adopts a previously unobserved short α-helix, while these three residues in the wild-type protein are located in a loop without secondary structure.

**Figure 2:**
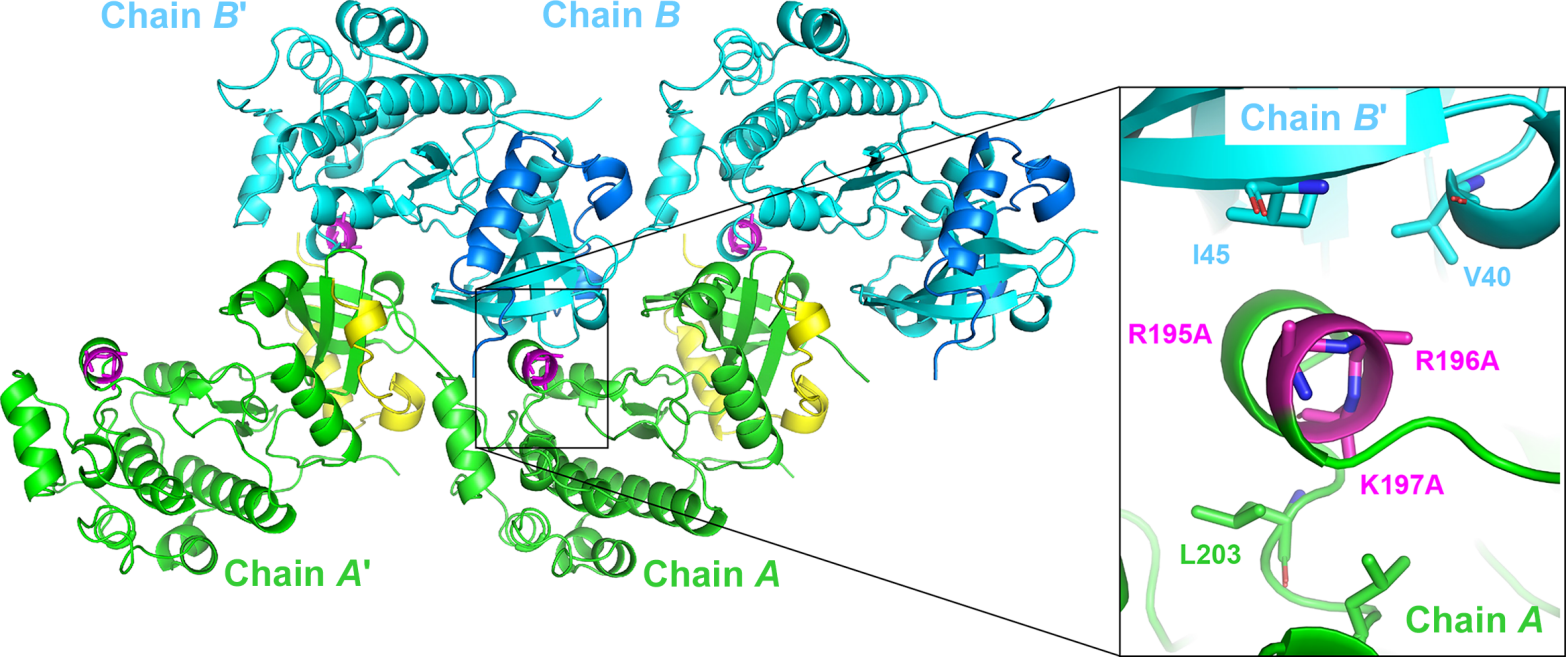
Crystal packing of the Aurora-C–INCENP complex. Chains *A* and *B* and symmetry mates chains *A*′ and *B*′ are shown in green and cyan, respectively. INCENP peptides are shown in yellow and blue. In both chains, the triple SER mutation R195A/R196A/K197A (shown with C atoms in magenta) is located in a short helix and contributes to a crystal contact which would not have been possible without the SER triple mutation.

Fig. 2 ▸ shows the crystal packing of the SER mutant form of Aurora-C and reveals that in both chains the new α-helix harbouring the SER triple mutation contributes to a crystal contact, with the side chains of R195A and R196A forming hydrophobic contacts to Ile45 (3.9 Å) and Val40 (4.4 Å), respectively, of a crystal neighbour, while the side chain of K197A contributes to intramolecular hydrophobic contacts (with Leu203 and Leu206) which help to stabilize the new α-helix to the body of the kinase domain.

As the introduction of the SER triple mutant was crucial to obtain crystals, we conclude that the triple mutation enabled the formation of the short helix in the activation segment, which stabilized the activation segment via interactions with the kinase domain and which additionally introduced a new crystal contact. Both the observed new hydrophobic crystal contact and the intramolecular interactions which pin this helix to the kinase core could not be established in the presence of the original arginine and lysine residues in positions 195–197.

In the two published structures of Aurora-C, the flexibility of the activation segment was probably overcome by using a longer and phosphorylated version of the INCENP peptide (834–903) and introducing a phosphoryl group at Thr198 in the activation segment, both of which were chosen to investigate the fully activated form of the Aurora-C–INCENP complex (Abdul Azeez *et al.*, 2019 ▸). In this system, Arg196 (part of our SER triple mutation) is engaged in three salt-bridge interactions: with the phosphoryl group at Thr198 of Aurora-C and with the phosphoryl groups at Ser893 and Ser894 of the INCENP peptide. These salt-bridge interactions stabilize the activation segment in a different way but, like our SER mutation, result in stabilization of this otherwise flexible region. In addition, by engaging Arg196 in an intermolecular salt bridge via the INCENP phosphoryl groups, the highly entropic surface cluster which we have removed by SER mutation is masked and can no longer negatively affect crystallization.

### 3.2. Structure of protein kinase IRAK4 via the SER approach

Our interest in IRAK4 as a target in the central nervous system and potentially dermatology had already started in 2003. At that time no structural data were available either for IRAK4 or for any other member of the IRAK family. IRAK4 is a serine/threonine kinase consisting of two domains: an N-terminal death domain (amino acids 20–104) and a C-terminal kinase domain (approximately amino acids 186–460). To support an HTS and a subsequent hit-to-lead campaign for identifying IRAK4 inhibitors, a complete gene-to-structure project was started. To increase the possibility of successfully obtaining a suitable crystallization system for repetitive protein–inhibitor complexes, we designed a set of 19 constructs (Table 4 ▸) addressing the following criteria. (i) Two different length variants (165–460 and 181–460) designed based on secondary-structure prediction tools as well as multiple sequence alignment of IRAK4 with known crystal structures of related kinases. (ii) Inactivating point mutations knocking out residues in the ATP-binding pocket which are involved in catalysis (constructs IRAK4_1, IRAK4_2, IRAK4_4 and IRAK4_5), with the aim of hindering potential autophosphorylation. (iii) A set of five double and triple SER mutations targeting surface clusters of lysine, arginine and glutamate residues, identified manually by inspecting the surface of a homology model of IRAK4 and introduced into the shorter and longer truncation variants used in this study (constructs IRAK4_6 to IRAK_15). In this SER approach, the target residues were only mutated to alanine, as recommended in the initial SER publication (Derewenda, 2004 ▸). (iv) The inclusion of pseudo-activating mutants of serine and threonine residues in the activation segment (constructs IRAK_17 to IRAK_20), which were designed to avoid inhomogeneous phosphorylation.

The short version of the inactive mutant construct IRAK4_5 did not yield any crystals at all. The long versions of the inactive mutant IRAK_2 and the SER mutant IRAK_9 resulted in crystalline material or even crystals, but were not further pursued because the initial diffraction was rather poor, at best to a resolution of only ∼10 Å.

Diffracting crystals were first obtained for IRAK4_12 and IRAK4_16. For IRAK_16 we obtained large hexagonal-shaped crystals using PEG 20 000 as a precipitant. The crystals diffracted to a maximum resolution of ∼3.5–4 Å at the BESSY synchrotron. With a *c* cell-axis length of ∼450 Å and rather poor diffraction quality, this crystal form was not further optimized. A second hexagonal crystal form was obtained using high amounts of PEG 3350 under slightly acidic conditions. These crystals diffracted routinely to up to 2.6 Å resolution. However, this crystal form showed high mosaicity and the *c* axis could often not be indexed in the diffraction images.

IRAK_12 immediately produced well diffracting crystals using high concentrations of ammonium sulfate at neutral pH. The crystals showed a tetragonal morphology and diffracted to 2.3 Å resolution in the tetragonal space group *I*4_1_22. The structure could not be solved using molecular replacement with the available related crystal structures at the time. SAD/MAD phasing was therefore performed using an osmium salt as a heavy-atom derivative (data not shown). After structure solution, we were able to build the C-terminal lobe of the kinase domain (without the activation segment) in the electron density, but surprisingly no electron density was observed for the complete N-terminal lobe, indicating that it was disordered in the crystal. We therefore stopped working on this short-length variant and switched back to construct IRAK_16.

A third, orthorhombic crystal form was then identified using ∼20% PEG 3500 as a precipitant and sodium tartrate as an additive. The crystals diffracted to a reasonable resolution of up to 2.4 Å in space group *I*222, with unit-cell parameters *a* = 86, *b* = 117, *c* = 141 Å. The structure was solved using the structure of IRAK_12 as a search model and this time large parts of the N-terminal lobe of the kinase were observed in the electron density and could subsequently be modelled. Despite this improvement, unambiguous modelling of the first HTS hits was hampered due to disordered regions in the active site of the kinase. At the same time, the first crystal structure of IRAK4 was published (Wang *et al.*, 2006 ▸), which was solved using an active wild-type construct (residues 154–460). The reported crystals grew under similar conditions to our shorter IRAK_12 variant, but again showed a different crystal packing in the monoclinic space group *C*2. Since Wang and coworkers showed that fully active protein can be crystallized with interpretable electron density in the active site, we switched to our kinase-active construct IRAK_6. Crystals were grown under two conditions (see Section 2) and crystallized in the orthorhombic space group *I*222, as we had previously observed for the IRAK_16 construct. In contrast to IRAK_16, the now fully active construct IRAK_6 showed well defined electron density in the active site for the HTS hits. We have recently reported the crystal structure determination of IRAK4, using construct IRAK_6, in complex with various small-molecule inhibitors (Bothe *et al.*, 2024 ▸).

**Figure 3:**
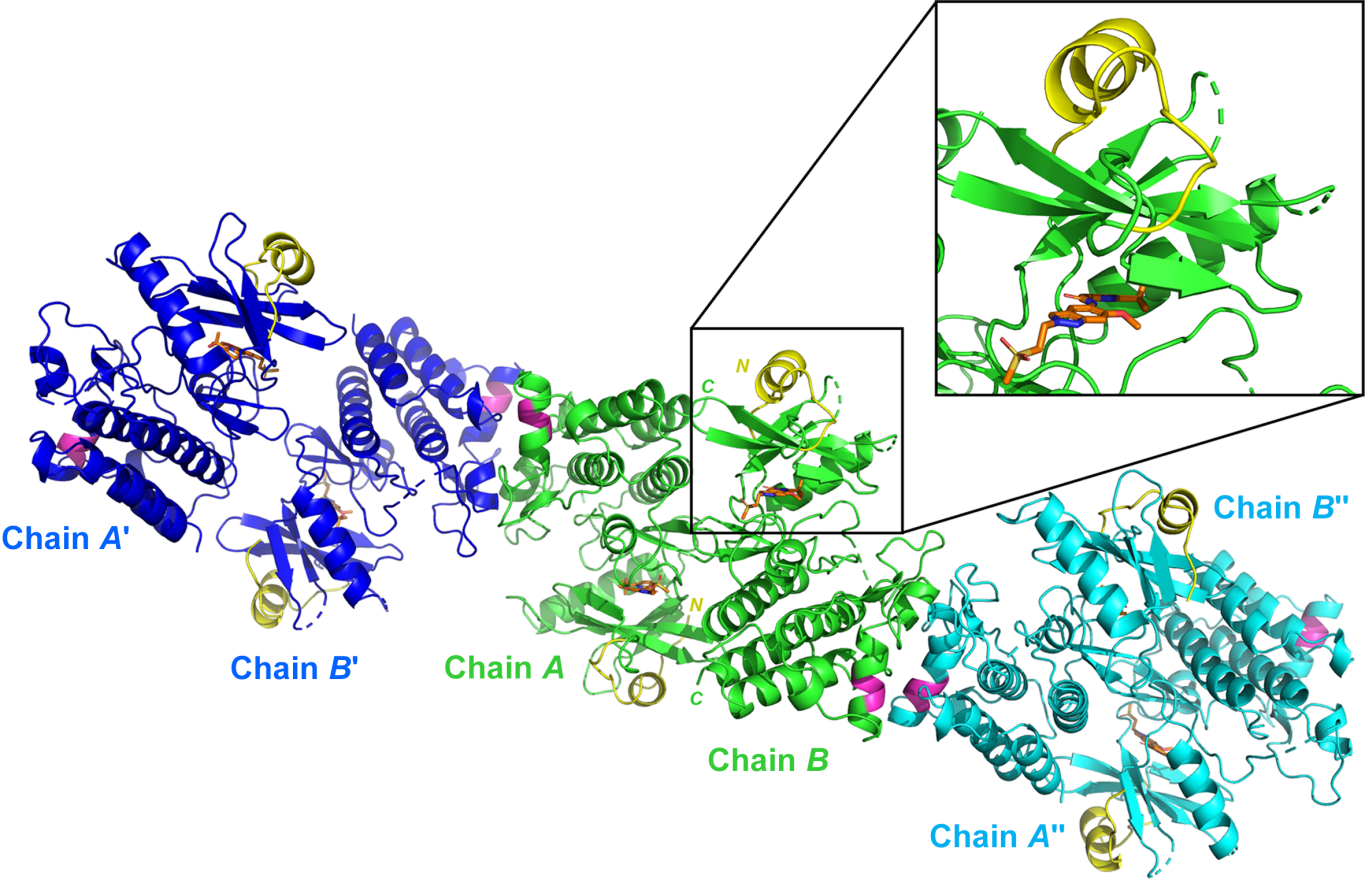
Crystal packing of the IRAK4 SER crystal form. The two chains of construct IRAK4_6 in the asymmetric unit of PDB entry 8br6 are shown in green ribbon representation. The position of the triple SER mutation K400A/E401A/E402A is highlighted in magenta. Two crystal neighbours in the vicinity of these SER mutations are depicted in cyan and dark blue. The N-terminal extension (residues 165–184), which is present only in the long constructs in Table 4 ▸, is shown in yellow. The co-crystallized inhibitor is shown in stick representation with C atoms in orange.

We expected that the three mutated amino acids might influence the crystal packing. However, analysis of the crystal packing showed that there are no interactions with neighbouring molecules in the cell. Overall, this observation is consistent with the observation that IRAK_16, although lacking the three SER mutations, crystallizes with an identical crystal packing. Moreover, in recent years many more crystal structures of IRAK4 in complex with inhibitors and featuring this *I*222 crystal packing have been published and none bear a SER mutation. Instead, we observe retrospectively that for IRAK4 selecting a subset of constructs with an extended N-terminus (marked as ‘long construct’ in Table 4 ▸) was of the utmost importance. In the crystal structure of the SER mutant IRAK_6 (PDB entry 8br6; Bothe *et al.*, 2024 ▸; Fig. 3 ▸) this N-terminal extension folds back onto the protein and forms an additional α-helix which most likely stabilizes the N-terminal lobe of the kinase. In a structure that we obtained from the construct without this extension (*i.e.* IRAK_12) the N-terminal lobe was completely disordered and the crystal form was therefore of no use for our HTS project.

### 3.3. Structure of a SER variant of BUB1

The BUB1 structural biology activities presented here also represent a complete gene-to-structure project which began at a time when there was no public domain information describing possible construct-design or crystallization strategies. Bioinformatic analysis clearly identified that the full-length protein, encoded by a total of 1085 residues, contained two terminal domains (an N-terminal Mad/Bub1 homology region and a C-terminal kinase domain) linked by extensive regions of predicted disorder. To support our interest in generating structural information for the kinase domain, initial protein-production and crystallization efforts led to the identification of the kinase-domain construct 726–1085, from which we were able to determine a ligand-complex crystal structure at 2.0 Å resolution (Siemeister *et al.*, 2019 ▸). Despite this success, experiments with other ligands were often hampered by both poor crystallization reproducibility and limited diffraction quality. With the aim of developing a more robust and better diffracting system, we designed a set of five second-generation constructs. Here, we specifically modified clusters of residues within the 726–1085 kinase-domain construct which, following the SER approach, feature residues with a high surface entropy, *i.e.* arginine, lysine, glutamate and glutamine residues (Table 5 ▸). These clusters were identified by manual inspection of the surface of a BUB1 homology model. In this SER study, we no longer exclusively mutated to alanine, but mixed in amino acids with relatively rigid but polar or partially polar side chains such as aspartate, threonine and tyrosine. This development reflected our own experience in previous SER projects and also subsequent literature reports (Cooper *et al.*, 2007 ▸) that the introduction of too many alanine residues often results in low solubility and that threonine and tyrosine can potentially contribute both hydrophobic and polar interactions to a possible new crystal contact. If such clusters contained cysteine residues, we mutated them to threonine or tyrosine to reduce the risk of unwanted oxidation or aggregation via disulfide bonds.

All second-generation SER mutants could be expressed and purified according to the protocols developed for the WT form of the protein (Siemeister *et al.*, 2019 ▸) and were subsequently tested in crystallization experiments. For all constructs a similar crystallization strategy was explored, whereby up to ten commercially available sparse-matrix screens and one fine screen around known crystallization conditions for the WT BUB1 protein were performed at both room temperature and 4°C.

**Figure 4:**
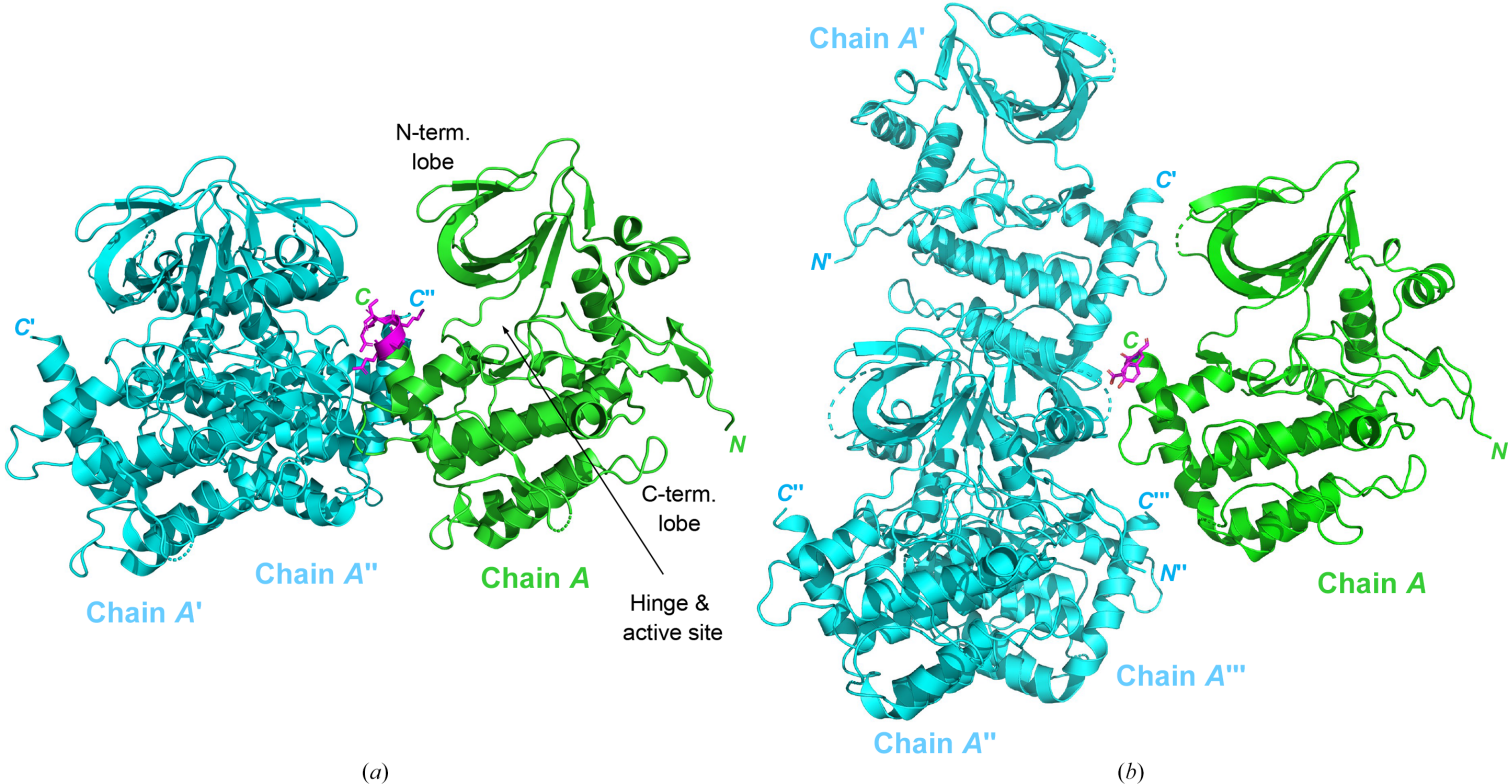
Crystal packing of the BUB1 SER crystal form. Structures of (*a*) WT BUB1 and (*b*) the BUB1_06 mutant, shown in green, with the mutated SER region (^1079^ECKRSRK^1085^→^1079^DYAPSYA^1085^) depicted in magenta. In both figures, crystallographic neighbours in the vicinity of the mutated site are depicted in cyan.

Focused crystallization screening around the conditions previously identified for BUB1^WT^ (Siemeister *et al.*, 2019 ▸) yielded a reproducible BUB1_06 crystallization system, with most crystals diffracting in the range 2–3 Å. The structure could be solved from one such data set by molecular replacement using the WT structure (PDB entry 6f7b) as a search model. The final structure contained one BUB1 chain in the asymmetric unit. The structure shares the same overall conformation as the nonmutated WT BUB1 protein (PDB entry 6f7b), namely the typical protein kinase fold with an N-terminal and a C-terminal lobe connected by a hinge region that flanks the ATP-binding site (Fig. 4 ▸*a*).

The packing of the BUB1_06 SER protein within the crystal differs significantly compared with that previously observed for the BUB1_01 WT protein (Fig. 4 ▸). This reflects the different crystal forms of the two proteins; the BUB1_01 WT construct crystallized in space group *P*2_1_2_1_2, whilst the BUB1_06 SER mutant crystallized in space group *P*2_1_. Compared with the WT BUB1 crystallization system, the crystallization of BUB1-^1079^DYAPSYA^1085^ was more reliable and a higher resolution could be achieved for different ligands without the need to screen an extensive number of crystals. As such, we were successful in obtaining a new BUB1 crystallization platform that was better suited to supporting drug-discovery activities. It was, however, not possible to retrospectively rationalize the molecular basis for these improved properties since the majority of the mutated ^1079^DYAPSYA^1085^ stretch of residues in the BUB1_06 SER mutant was disordered and could not be unambiguously modelled. This highlights the complexity of the protein crystallization process and suggests that the mutated surface residues may also promote improved crystallization properties by influencing the early stages of crystal formation, rather than specific stabilizing interactions in the final crystal packing.

### 3.4. Crystallization of the KRAS^G12C^–SOS1 complex

**Figure 5:**
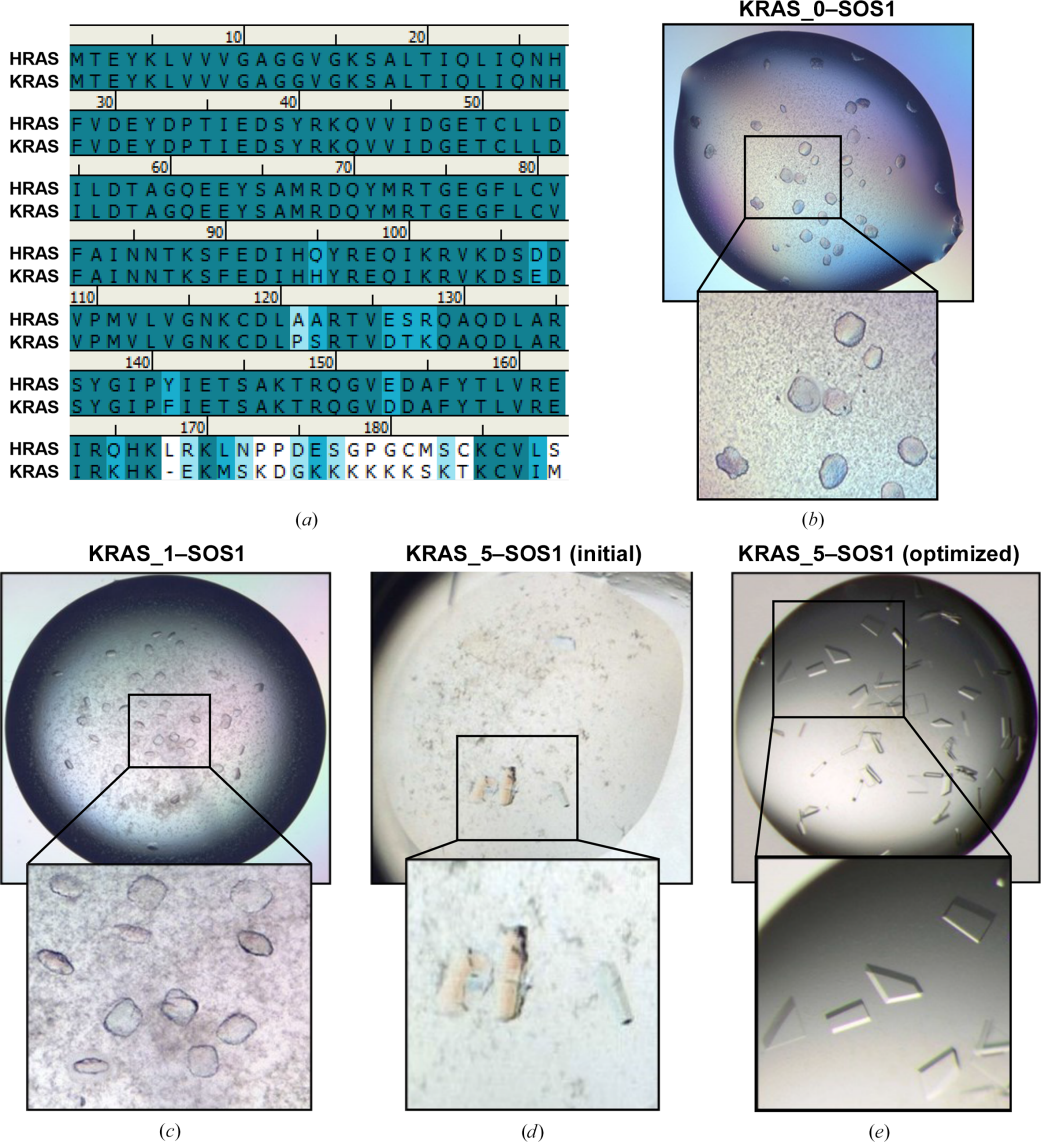
Crystallization of the KRAS^G12C^–SOS1 complex. (*a*) Sequence alignment of human KRAS (UniProt ID P01112) and HRAS (UniProt ID P01116_2). (*b*) Initial crystals obtained using KRAS^G12C^ without any further surface modification (construct KRAS_0). The crystals have a diameter of up to 60 µm. (*c*) KRAS^G12C^–SOS1 crystals obtained with construct KRAS_1 (maximum diameter 50 µm). (*d*) Initial KRAS^G12C^–SOS1 crystals obtained with the construct KRAS_5 (largest rod-shaped crystal 40 × 150 µm). (*e*) Optimized KRAS^G12C^–SOS1 crystals with construct KRAS_5 (largest plates ∼80 × 80 × 30 µm).

We have recently reported a fragment-screening campaign for binders and stabilizers of the KRAS^G12C^–SOS1 complex as starting points for inhibitors of the KRAS^G12C^–SOS1 interaction as novel anticancer drugs (Hillig *et al.*, 2019 ▸). For this fragment screen, we originally envisaged following the purification and crystallization approach reported previously for the HRAS–SOS1 complex (Boriack-Sjodin *et al.*, 1998 ▸). However, initial experiments using the same SOS1 construct (residues 564–1049) and just replacing HRAS (WT) with KRAS^G12C^ resulted in poorly grown crystals (Fig. 5 ▸*b*). Despite extensive optimization efforts, these could not be optimized into single crystals and diffraction was limited to about 5 Å. In contrast, we were able to reproduce the reported well diffracting HRAS–SOS1 crystals (Boriack-Sjodin *et al.*, 1998 ▸), indicating that the sequence differences between KRAS and HRAS may be responsible for the different crystallization outcomes.

Based on this observation, we explored two alternative surface-mutation strategies in parallel. In the first, we followed the classical SER approach and selected surface residues which, based on the available structure of KRAS and HRAS–SOS1, formed clusters with high surface entropy on the surface of KRAS. These were identified by manual inspection of the solvent-accessible surface of KRAS (PDB entry 4dsu) superimposed onto the co-complex structure of HRAS–SOS1 (PDB entry 1bkd). In the second, and ultimately successful, strategy we selected residues that differed between KRAS and HRAS (see the sequence alignment in Fig. 5 ▸*a*) and were involved in crystal contacts in the well diffracting crystal form published for HRAS–SOS1 (PDB entry 1bkd). These were mutated to make KRAS^G12C^ more HRAS-like (the ‘KRAS-to-HRAS’ approach). In both approaches care was taken to not change any KRAS surface residues which contribute to the SOS1-binding epitope (as predicted from the SOS1-binding epitope in the structure with HRAS; PDB entry 1bkd). Finally, to test whether the longer C-terminus of KRAS (1–169) compared with HRAS (1–166) may have caused the crystal-growth problems, we also tested a KRAS construct with a shorter C-terminus with and without surface mutations. All eight mutants are summarized in Table 6 ▸.

All eight KRAS^G12C^ surface mutants were expressed on a small scale, and in test expressions all eight proteins showed solubility comparable to the wild-type protein. We prioritized constructs KRAS_1, KRAS_5 and KRAS_8 as representatives of the two approaches and of the longer and shorter C-terminus. For all three the complexes with SOS1 were generated and crystallization screens were set up. We obtained no crystals when using KRAS_8 (SER strategy) and only poorly grown crystals using KRAS_1 (SER and KRAS-to-HRAS strategy) (Fig. 5 ▸*c*). Interestingly, the latter crystals displayed a very similar morphology to KRAS^G12C^–SOS1 crystals that did not contain any surface-residue modifications (Fig. 5 ▸*b*).

**Figure 6:**
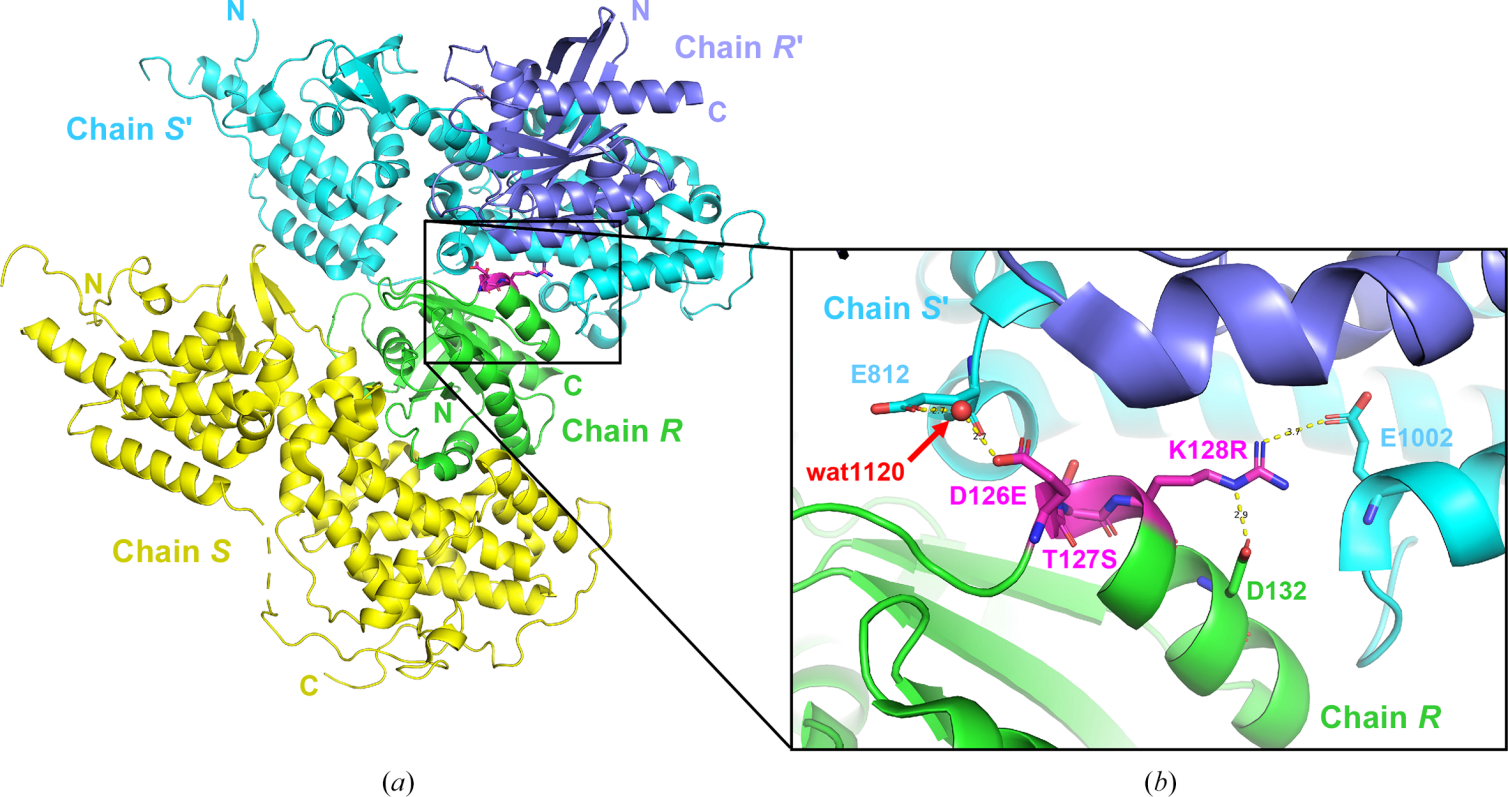
Crystal packing of the KRAS^G12C^–SOS1 complex crystal form. (*a*) Crystal packing of the complex with KRAS_05 [ribbon representation with KRAS_05 (chain *R*) in green and SOS1 (chain *S*) in yellow]. The triple KRAS-to-HRAS mutation D126E/T127S/K128R is shown in magenta (stick representation). Two adjacent symmetry mates are depicted in cyan (SOS1 crystal mate, chain *S*′) and slate blue (KRAS crystal mate, chain *R*′). The enlarged view in (*b*) shows the crystal-contact interactions formed by two residues of the triple KRAS-to-HRAS mutation.

Crystals of the complex of SOS1 with KRAS_5, following the KRAS-to-HRAS approach, showed a new morphology (Fig. 5 ▸*d*) and could quickly be optimized into single crystals that diffracted to better than 2.3 Å resolution (Fig. 5 ▸*e*). This system allowed us to solve several co-crystal structures in complex with fragment hits (Hillig *et al.*, 2019 ▸). The obtained crystal form (represented by PDB entry 6epl) belongs to space group *I*422, with unit-cell parameters *a* = *b* = 150, *c* = 202 Å, and is thus isomorphous with the HRAS–SOS1 crystal structure with PDB code 1bkd (*I*422, with unit-cell parameters *a* = *b* = 143, *c* = 208 Å). The three point mutations D126E, T127S and K128R contribute to a crystal contact (Fig. 6 ▸): Glu126 forms a water-bridged hydrogen bond to Glu812 of an adjacent SOS1 molecule (2.7 Å/2.7 Å). This contact would not have been possible with the shorter side chain of an aspartate as in the wild-type KRAS protein. The second mutation (T127S) is not directly involved in a crystal-contact interaction. For the third mutation, K128R, the side chain of Arg128 is stabilized in its position by an intramolecular hydrogen bond to Asp132 (2.9 Å) and donates a (weak) hydrogen bond (3.7 Å) to Glu1002 of the same SOS1 symmetry mate. Again, the original lysine in this position in wild-type KRAS would have been too short to form this contact. It is worth noting that both of these ‘KRAS-to-HRAS’ mutations run against the SER principle and either bring in a higher entropy residue (D126E) or keep a high-entropy residue (K128R). However, in sum they still helped this crystal lattice to form.

## 4. Summary and outlook

In this contribution, we have presented case studies exemplifying our typical approaches for enabling robust crystallization platforms for challenging target proteins. Such strategies may support both the crystallization of proteins for which no conditions can be identified as well as the optimization of poorly reproducible, or poorly diffracting, crystals. The presented case studies share the common theme that the targeted modification of specific surface residues supported improved protein crystallization properties. Typically, the design and introduction of the mutation(s) builds on previous knowledge of the optimal expression and purification strategies for the native protein sequence.

These case studies were selected to represent a broad range of different factors that may be considered when designing surface-residue modifications. Most importantly, the mutated residues should not influence positions of functional importance and should not be in or adjacent to the binding site that is the focus of the discovery/optimization program. Different design strategies may then be considered, including the reduction of secondary-structural conformation heterogeneity, the reduction of side-chain conformational heterogeneity and the engineering of new crystal contacts.

Highly mobile structural elements in proteins often play a central role in the regulation of the activity of the protein. Despite their biological importance, this flexibility may present a challenge in crystallization experiments. The kinase-activation segment, as shown by the Aurora-C example in this manuscript, is a typical example of such a highly mobile structural element. The common approach of addressing the phosphorylation status of this region did not support crystallization. However, identification of an SER-triple mutation which simultaneously stabilized the conformation of the activation segment contributed to the successful crystallization, confirming that it was indeed the high flexibility in this region that was hampering target crystallization. The triple SER mutation allowed the intrinsically very flexible kinase-activation segment to adapt a new and partly surface-exposed helix. The three new alanine surface residues contributed both to the anchoring of this helix to the protein core as well as to the formation of a new crystal contact.

The second strategy, namely the reduction of side-chain conformational heterogeneity or surface-entropy reduction (SER), is well suited to almost all protein targets, and has become a valuable tool which we routinely test in our second-generation construct-design cycles. In addition to the identification of appropriate sites to modify, the selection of which residue to mutate to is also an important consideration. In line with literature reports on systematic mutations to either alanine (Derewenda, 2004 ▸) or to tyrosine and threonine (Cooper *et al.*, 2007 ▸), we have also observed success with a selection of different SER strategies. Mutation of high-entropy surface clusters to alanine in both Aurora-C and IRAK4, as well as the more unusual BUB1 strategy, in which three of five high-entropy surface residues were replaced with non-alanine residues (namely tyrosine and serine), highlights the spectrum of possibilities. Indeed, other examples have also been described, for example the recently reported robust protein kinase PLK1 crystallization system in which two adjacent surface lysine residues were mutated to aspartate and alanine (Hillig, manuscript in preparation). Intriguingly, whilst the theory of how such mutations support the crystallization of proteins is well described, our retrospective analysis of both IRAK4 and BUB1 did not identify any strong features within the structures and crystals that could be directly attributed to the improved crystallization properties.

The third strategy presented in this paper was rational crystal-contact engineering in the context of establishing a KRAS–SOS1 crystallization system suitable for the characterization of fragment hits. Crystal-contact epitopes identified in a well crystallizing close relative (here HRAS in the HRAS–SOS1 complex) were transferred into the less well crystallizing KRAS–SOS1 complex. This KRAS-to-HRAS approach was indeed successful and allowed us to establish a robust and well diffracting KRAS–SOS1 system. Retrospective analysis highlighted that the mutation did indeed facilitate a new crystal contact, as observed in the related HRAS–SOS1 crystal structure. Such opportunities are highly dependent on the availability of related structures with sufficient sequence and structural homology.

The case studies presented here highlight the broad surface-mutagenesis toolbox that can be explored to establish robust crystallization systems for challenging targets. Whilst there is no one-size-fits-all solution, experience with the different strategies allows an expert to design a subset of tailor-made mutation constructs that, with the help of high-throughput protein-production and crystallization platforms, can be evaluated for improved crystallization properties. Interestingly, for especially challenging targets a combination of multiple independent strategies may be required, with the cumulative result that the target crystallization can be enabled. In addition to a SER triple mutation, the Aurora-C crystallization additionally required the presence of both a stabilizing protein (INCENP) and a high-affinity inhibitor.

In conclusion, surface-mutagenesis strategies are a powerful method for the establishment of robust crystallization systems. They are a routine component of our crystallization platform and have allowed us to enable structure-based drug discovery with many therapeutically interesting targets.

## Supplementary Material

PDB reference: Aurora-C with surface-entropy reduction mutation in complex with INCENP peptide, 9esa

## Figures and Tables

**Table 1:** Expression constructs for protein kinase Aurora-C

Construct	Termini	Surface mutations	Rationale	Crystallization outcome
AurC_1	13–301	None (wild type)	Length variant	No crystals
AurC_2	13–309	None (wild type)	Length variant	No crystals
AurC_3	28–301	None (wild type)	Length variant	Not tested (poor expression)
AurC_4	28–309	None (wild type)	Length variant	Not tested (poor expression)
AurC_5	35–301	None (wild type)	Length variant	Not tested (poor expression)
AurC_6	35–309	None (wild type)	Length variant	Not tested (poor expression)
AurC_7	13–309	S193D/T198D/T202D	Activated state mimic	No crystals
AurC_8	13–309	R195A/R196A/K197A	SER	Crystal structure (with INCENP)

**Table 2:** Data-collection and processing statistics for the Aurora-C–INCENP complex Values in parentheses are for the outer shell.

PDB code	9esa
Diffraction source	Rigaku MicroMax-007 HF
Wavelength (Å)	1.5418
Temperature (K)	100
Detector	Rigaku R-AXIS IV++
Crystal-to-detector distance (mm)	200
Rotation range per image (°)	0.5
Total rotation range (°)	140
Exposure time per image (s)	360
Space group	*C*222_1_
*a*, *b*, *c* (Å)	79.28, 79.49, 265.24
α, β, γ (°)	90, 90, 90
Mosaicity (°)	0.7
Resolution range (Å)	19.87–2.80 (2.90–2.80)
Total No. of reflections	100398
No. of unique reflections	21012
Completeness (%)	99.6 (99.9)
Multiplicity	4.8 (5.1)
〈*I*/σ(*I*)〉	6.1 (2.3)
*R*_merge_	0.126 (0.350)
*R*_r.i.m._	0.142 (0.390)†
Overall *B* factor from Wilson plot (Å^2^)	65.1

† The redundancy-independent merging *R* factor *R*_r.i.m._ was not available and was estimated by multiplying the conventional *R*_merge_ value by the factor [*N*/(*N* − 1)]^1/2^, where *N* is the data multiplicity.

**Table 3:** Structure solution and refinement of the Aurora-C–INCENP complex Values in parentheses are for the outer shell.

Resolution range (Å)	19.87–2.80 (2.87–2.80)
Completeness (%)	99.3 (99.9)
No. of reflections, working set	19891
No. of reflections, test set	1075
Final *R*_cryst_	0.227 (0.364)
Final *R*_free_	0.294 (0.395)
Cruickshank DPI	0.433
No. of non-H atoms	
Aurora-C, chain *A*/*B*	2252/2275
INCENP, chain *C*/*D*	352/358
Ethylene glycol	4
Water	56
Total	5297
R.m.s. deviations	
Bond lengths (Å)	0.004
Angles (°)	1.279
Average *B* factors (Å^2^)	
Aurora-C, chain *A*/*B*	74.0/74.5
INCENP, chain *C*/*D*	77.3/78.3
Ethylene glycol	85.8
Water	52.9
Total	74.5
Ramachandran plot	
Most favoured (%)	90.9
Allowed (%)	7.9

**Table 4:** Expression constructs used for protein kinase IRAK4

Construct	Termini	Surface mutations	Rationale	Crystallization outcome
IRAK4_1	165–460	K213A, K214A	Kinase-inactive mutant, long construct	Not tested
IRAK4_2	165–460	D329N	Kinase-inactive mutant, long construct	Crystals that did not diffract well
IRAK4_3	181–460		Wild type, short construct	Not tested
IRAK4_4	181–460	K213A, K214A	Kinase-inactive mutant, short construct	Not tested
IRAK4_5	181–460	D329N	Kinase-inactive mutant, short construct	No crystals obtained
IRAK4_6	165–460	K400A, E401A, E402A	SER, long construct	Final construct, 2.1–2.5 Å
IRAK4_7	165–460	E406A, E407A, K408A	SER, long construct	Not tested
IRAK4_8	165–460	K416A, K417A	SER, long construct	Not tested
IRAK4_9	165–460	E439A, K440A, K441A, K443A	SER, long construct	Crystals with poor diffraction
IRAK4_10	165–460	K448A, K449A	SER, long construct	Not tested
IRAK4_11	181–460	K400A, E401A, E402A	SER, short construct	Not tested
IRAK4_12	181–460	E406A, E407A, K408A	SER, short construct	Crystals, solved; N-terminal lobe disordered
IRAK4_13	181–460	K416A, K417A	SER, short construct	Not tested
IRAK4_14	181–460	E439A, K440A, K441A, K443A	SER, short construct	Not tested
IRAK4_15	181–460	K448A, K449A	SER, short construct	Not tested
IRAK4_16	165–460	T351D, T352D	Pseudo-active mutant, long construct	Crystals, solved
IRAK4_17	181–460	T351D, T352D	Pseudo-active mutant, short construct	Not tested
IRAK4_18	181–460	T324D, T351D, T352D	Pseudo-active mutant, short construct	Not tested
IRAK4_19	181–460	T324D, T345A, S346A, T351D, T352D	Hyper-pseudo-active mutant, short construct	Not tested

**Table 5:** Expression constructs used for protein kinase BUB1

Construct	Termini	Surface mutations	Rationale	Crystallization outcome
BUB1_1	726–1085	None	WT (Siemeister *et al.*, 2019 ▸)	Crystals that did not work with certain inhibitors
BUB1_2	726–1085	K815A, Q816D, K817A	SER	No crystals
BUB1_3	726–1085	E886N, K887A	SER	No crystals
BUB1_4	726–1085	E931D, Q932A, D933Y, D934A, E935S	SER	No crystals
BUB1_5	726–1085	K965A, C966T, E967D	SER	No crystals
BUB1_6	726–1085	E1079D, C1080Y, K1081A, R1082P, R1084Y, K1085A	SER	Final construct, structures to 2–3 Å resolution

**Table 6:** Expression constructs used for KRAS All constructs contained the oncogenic mutation G12C and the technical mutation C118S (Sun *et al.*, 2012 ▸).

Construct	Termini	Surface mutations	Rationale	Crystallization outcome
KRAS_0	1–169	None	Wild type, long construct	No single crystals, only 5 Å resolution
KRAS_1	1–169	E107D	SER and KRAS-to-HRAS	No single crystals
KRAS_2	1–169	E107A	SER	Not tested
KRAS_3	1–169	K128R	KRAS-to-HRAS	Not tested
KRAS_4	1–169	K128Y, R135A	SER	Not tested
KRAS_5	1–169	D126E, T127S, K128R	KRAS-to-HRAS	Well diffracting crystals, final construct
KRAS_6	1–166	None	Wild type, short construct	Not tested
KRAS_7	1–166	K165Q	KRAS-to-HRAS	Not tested
KRAS_8	1–166	K165A	SER	No crystals
